# Cutaneous Diseases

**Published:** 1875-11

**Authors:** 


### III. Cutaneous Diseases.

1. *Elephantiasis Arabum.* Fayrer, Jos., C. S. I., M.D., F.R.S.E. (*Practitioner*, Aug., 1875.)

The disease is defined to be a non-contagious malady, endemic in certain localities, generally intertropical and near the sea-coast, characterized by recurrence of febrile paroxysms, attended by inflammation and progressive hypertrophy of the integument and areolar tissue, chiefly of the extremities and genital organs; and occasionally by swelling of the lymphatic glands, enlargement and dilatation of the lymphatics, in some cases by the coexistence of chyluria and the presence in the blood of certain nematode hæmatozoa; the hypertrophy of the integument resulting in enormous enlargements of the scrotum or labia, accompanied by an albuminous deposit in the cells of the areolar tissue, and by degeneration of the muscular and osseous tissues.

The outgrowths are viewed as local expressions of a constitutional disease, cases undoubtedly occurring where the reverse is true, as in Europe and elsewhere. Malarial influences are presumed to play an important part. Both sexes, and persons of all ages and conditions of life, are liable to the affection, which is, however, most common in adult and middle life. Sometimes the general health seems unimpaired, while again there is recurrence of fever once or twice in the month. The average duration of the disease, in 636 cases, was 11½ years, the youngest patient being nine years of age and the oldest eighty. It had, apparently, but little tendency to shorten the duration of life, while not only in mechanical but in general cachectic influence, it diminished the power of procreation.

The author agrees with Lewis in referring to a common etiological origin, elephantiasis, chyluria and disordered conditions of the lymphatic system. The last named writer is disposed to believe that the *Filaria sanguinis hominis*, a small nematode entozoon, is intimately associated with the three classes of disorders when occurring in tropical latitudes. The parasite is enclosed in a tubular sheath, within which it elongates or shortens itself, having the average diameter of a blood corpuscle and an average length forty-six times that of its greatest width. It is smaller but more allied in embryo to the *Filaria medinensis* than to the larvæ of *Trichina spiralis*.

The microscope disclosed, chiefly, an hypertrophied areolar tissue in the sections examined.

2. *Tar in Psoriasis.* M'Call Anderson. (*Brit. Med. Jour.*)

Objection is made to the conclusion reached by Balmanno Squire that "tar taken internally has no effect in curing psoriasis." After many years of experience the author has come to regard it in the light of one of the most valuable remedies we possess in the treatment of that disease. Not only in mild, but in obstinate cases, after long courses of arsenic have been vainly tried, is its use beneficial. He ordinarily begins with two minims, three times a day, in a teaspoonful of treacle, and, if necessary, increases the dose to half a teaspoonful, or even more. The smaller dose is advised at first, since in certain cases there is intoleration of the remedy, and in others, digestive disturbances, fever and bright red exanthem.

He also testifies to the virtues of the remedy in catarrh of the bronchial tubes (Ringer) and in chronic affections of mucous membranes, and deems it remarkable that a remedy once so highly esteemed, should, as an internal medicine, have fallen into such disrepute in our own time.

3. *Non-specific Rupia prominens.* GODON. (*Medical Record*, Sept. 4.)

A conical crust or scab on the left side of the external surface of the prepuce, was observed in a patient 23 years of age. Dark blue rings, with a lighter-colored interspace, were surmounted by a thick, hard, dark, bluish cap. The base secreted a mass of yellow pus, confined and supported by a thin film. At right angles to the base, ran darkish streaks on the film, through which pus oozed. The base was surrounded by a pinkish and inflamed margin, about a quarter of an inch or less in width, which did not implicate the healthy skin. Vertically, the crust measured three-fifths of an inch; the circumference, one inch and nine-tenths. There had been no sexual intercourse for two months and a half; one year previous there had been gonorrhœa. The patient was fair, pale, with a large head and prominent forehead, and was evidently scrofulous. There had been pre-existent rheumatism. History and signs of syphilis were all wanting. On the removal of the crust, sanious, yellowish-gray pus was found in quantity beneath.

The case is interesting in view of the fact that dermatologists are not agreed as to whether rupia is, or is not, an exclusively syphilitic lesion.

4. *Multiple Cutaneous Sarcomata.* VIDAL. (*Le Progrès Médical*, July 31, 1875.)

A patient was exhibited to the Biological Society, upon whose skin reddish maculæ first appeared and gradually became converted into ulcerative tumors, similar to others which had previously existed upon the glans penis and prepuce. After division they assumed the phases of repair, but when subjected to friction they became covered with a dense epidermis. The patient had survived the two or three years which constitute the usual duration of the disease, and his general health was unimpaired. Under examination, the tumors exhibited the characteristic structure of fasciculated sarcoma.

5. *Local Treatment of Cutaneous Diseases.* BRAME of Tours. (*Le Progrès Médic.*, Aug. 28, '75.)

All cutaneous disorders are local and curable by local applications; chiefly by coal-tar, the iodide of silver and white precipitate. Tinea is relieved without resorting to epilation. The author was less successful in psoriasis. He believes that all these disorders result from chronic superficial lymphangitis, his conclusions being based upon examination of 1,500 cases. Verneuil, in commenting upon the above, properly insisted that, if all the diatheses were to be excluded from the etiology of cutaneous diseases, syphilis should certainly be excepted.

6. (A) *Goa Powder and Poh di Bahia in Eczema Marginatum.* THIN. (*The Practitioner*, July, 1875.)

(B) *Goa Powder.* FERRAND. (*La France Médicale*, Sept. 1, 1875.)

(A) Köbner discovered the tricophyton tonsurans in the epidermic scales of eczema marginatum, and considered the disease to be ringworm modified by its occurrence in a humid and heated locality. Hebra, while admitting the occasional existence of the parasite, describes eczema marginatum as characterized by a hard, raised, infiltrated margin, most commonly curving downwards on the inner surface of the thigh, or backwards over the hips. It may also be found independently elsewhere. It is easily distinguished from ringworm by its objective features, and the results of treatment.

Dr. Thin, while in Shanghæ, frequently found ordinary ringworm disappear under the use of simple remedies, while the marginate eczema required months of treatment; and then almost invariably there was relapse. He distinguishes a wide difference between the two affections, and considers that the rarity of the last named disease in England and throughout Europe, has contributed to the adoption of Köbner's view. In China, practitioners have no hesitation in making a like distinction; eczema marginatum with them resisting anti-parasitic treatment.

Dr. Henderson, of Shanghæ, informed him by letter, that the profession in China were at last able to cope with the disease by the aid of a secret remedy, Goa powder, which was speedily becoming popular. Dr. Thin gives interesting details, in the case of a gentleman who got Burmese or Chinese ringworm in Singapore, and was relieved by both the Poh di Bahia and the Goa powder. A specimen of the drug obtained in London did not answer the purpose; it was supposed to have lost its efficiency from age. This patient preferred the Poh di Bahia to the other, as less irritating and more speedy in its effects.

(B) Ferrand reports that Goa powder first appeared in Europe in 1866 or 1867, and that Blanc, a surgeon of the English army in India, called attention to the value of the remedy in tinea circinata. From him M. Gubler obtained a specimen of the drug which he has analyzed.

Acording to Attfield, chrysophanic acid constitutes nearly 80 per cent. of the powder; the remainder being composed of two principles, one bitter, the other sweet and resinous. It is generally supposed to be the powder of a lichen exported from Mozambique. Others believe it to be the dried product of one of the leguminosæ. Gubler dissents from these opinions.

The specimen obtained from Blanc, and that exhibited to the Pharmaceutical Society by Limousin, were resinous to the touch, yellowish gray, insoluble even in warm water which they slightly discolored, and in muriatic acid. They were readily dissolved in benzine, ether and chloroform. Solutions in the two last named vehicles, were of a beautiful emerald green hue. Alkalies—and the fixed alkalies in particular—readily dissolved the powder. The solution, which is at first brown, assumes, after contact with the air, a superb purplish red color, the ammoniacal solution slowly turning to a violet shade. The dry powder warmed in a tube volatilizes, the vapor condensing in minute crystals upon the glass. If the heat becomes excessive, a black residuum is left after the disengagement of empyreumatic products. The residuum afterward is readily dissolved in chloroform, with production of a green shade. These are the reactions of chrysophanic acid.

Under the microscope, resinoid concretions of irregular concave or mammillated surface, and of various thickness, showed evidence of mechanical fracture. A superficial violet-colored pellicle, with distinct points of metallic lustre, covered an inner, pale yellow layer of friable, homogeneous and delicate texture. There was no debris of tissue, vessels or histological elements.

The woody fragments were covered with a dense layer of amorphous, resinoid substance, occasionally appearing like a delicate crystalline crust of a bright yellow shade. Microscopically, the prismatic, elongated or rhomboidal crystals all resembled those of chrysophanic acid, with which evidently the wood was not only covered but penetrated, in ligneous fibres and cellules.

In short, the two specimens were identical in composition, and, though it is not certain, it is at least probable that Goa powder is derived from a species of cassia—perhaps a senna—which is known to contain chrysophanic acid, and is used in the Orient to relieve various parasitic cutaneous diseases.

